# 

**Published:** 1916-08-19

**Authors:** 


### CONTENTS.

| | PAGE |
|---|---|
| **Free Drugs and State Control** ... ... ... | 461 |
| **Local War Committees and Local Authorities** | 462 |
| **Hospital and Institutional News** ... ... ... | 463 |
| Whistling for "Taxis" in London—A New Australian War Hospital—An Exclusive Suburb and Disabled Soldiers—Should the Churches be Converted into Hospitals?—Clubs and Hotels as Hospitals—A Joke in a Will—The East Sussex Hospital: A Big Miscalculation—Gloucester Red Cross Hospital's New Wing—The Harold Fink Hospital—The Treatment of Camp Workers—The Wounded at Essex County Hospital—The New Hull Sanatorium—Sketches in Hospital—Compulsory Anæsthesia in France—Send One Child to the Country—Fifty Millions in War Gifts—Increasing Duties of Medical Officers of Health—Should Saturday Fund Subscriptions be Raised?—The Case for Butter in Phthisical Wards—This Week's Drug Market. | |
| **National Insurance and the Voluntary Hospitals** ... ... ... ... ... ... | 467 |
| The Case of St. Bartholomew's Hospital. | |
| **A New Treatment for Infected Wounds** ... | 468 |
| Bismuth and Iodoform Paste. | |
| **Our Hospitals and the National Relief Fund...** | 469 |
| The Point of View of the Special Hospitals: Metropolitan Hospitals—Provincial Hospital Managers' Views. | |
| **Human Temperaments** ... ... ... ... | 471 |
| IX.—The Envious Temperament. | |
| **Parliament and the Profession** ... ... ... | 472 |
| **The Matrons' and Sisters' Department** ... | 473 |
| NOT "Good Enough for the War." | |
| Round the Hospitals. | |
| Independence for Poor-Law Nurses—Nurses' Off-Time in Daylight—Morning Classes for Probationers — Manners and Massage—Nurses, Patients, and Conversation. | |
| **National Poor-Law Officers' Association** ... | 475 |
| Grave Crisis for Poor-Law Nurses. | |
| **The National Medical Union and the Insurance Acts** ... ... ... ... ... ... ... | 476 |
| **Metropolitan Hospital Sunday Fund** ... ... | 477 |
| Distribution of Awards. | |
| **Gifts to Hospitals** ... ... ... ... ... ... | 480 |
| **Personalia...** ... ... ... ... ... ... ... | 480 |
| **Matrons' and Sisters' Appointments** ... ... | 480 |
| **The Institutional Worker** ... ... (Suppl.) | 1 |
| Labour-Saving Appliances. | |
| Question for August. | |
| Questions Invited. | |
| Enquiries and Answers. | |
| The Sick and in Need. | |
| The Housekeepers' Department. | |
| Miscellaneous. | |

